# Malignant Large Bowel Obstruction Masquerading as Benign Inflammatory Disease in a Young Male: A Cautionary Tale of Diagnostic Delay

**DOI:** 10.7759/cureus.116253

**Published:** 2026-09-14

**Authors:** Varsha G Baker, William G Baker, Sindhu Para, Greg Crisafulli, Meghan Daly, Aditi Jalla

**Affiliations:** 1 Colorectal Surgery, University of Florida College of Medicine - Jacksonville, Jacksonville, USA; 2 Colorectal Surgery, St. George's University, True Blue, GRD; 3 Surgery, St. Joseph's Regional Medical Center, Paterson, USA; 4 Critical Care Surgery, University of Florida College of Medicine - Jacksonville, Jacksonville, USA

**Keywords:** colon mass, hostile abdomen, large bowel obstruction, palliative surgery, peg tube

## Abstract

We present the case of a 35-year-old male with an acute large bowel obstruction (LBO) secondary to a sigmoid mass, which was proximally diverted with a loop colostomy at an outside hospital. The mass could not be resected, and tissue could not be obtained for pathological diagnosis because of ongoing concerns about infection. Ultimately, the patient was found to have a frozen, hostile abdomen, and a palliative percutaneous endoscopic gastrostomy tube was placed for venting. We discuss surgical management options, pathology, and a brief review of the literature.

## Introduction

Small bowel obstruction (SBO) is a common surgical emergency caused by mechanical or functional disruption of intestinal flow. Mechanical SBO is most frequently due to intra-abdominal adhesions, especially in patients with prior abdominal surgery. Other causes of SBO include malignancies, hernias, inflammatory conditions, and less commonly, radiation injury or endometriosis. Functional obstruction (ileus), on the other hand, results from impaired motility and is often related to metabolic disturbances, medications, or systemic illness. Lastly, adhesive SBO (ASBO) represents a particularly challenging subset, as adhesions can be dense, difficult to dissect, and may mask underlying pathology. Differentiating benign adhesive obstruction from more serious pathology such as malignancy is often complex, especially in the absence of prior abdominal surgery or definitive imaging findings [1-5].

Laboratory tests, including a complete blood count (CBC), electrolytes, renal function tests, arterial blood gas (ABG), lactate levels, amylase/lipase, and blood cultures, are commonly used to support the evaluation of suspected SBO. However, while these tests can provide helpful clinical information, imaging is essential for definitive diagnosis [1,2]. The initial imaging modality of choice is typically an abdominal radiograph, which may reveal dilated loops of small bowel, air-fluid levels, absence of gas in the colon, or signs of perforation. However, a CT scan is considered the gold standard for diagnosing SBO. It provides detailed information, including transition points, mesenteric stranding, and potential complications such as strangulation or bowel ischemia [1,2]. Management of SBO depends on the underlying cause and clinical context. All patients require prompt fluid resuscitation. For simple mechanical SBO without signs of ischemia or peritonitis, conservative management is appropriate. This includes bowel rest (NPO), nasogastric decompression if necessary, serial abdominal examinations, frequent laboratory monitoring, and vital sign assessment. Timely diagnosis and intervention are critical. Delays can lead to serious complications such as strangulation, bowel ischemia, and sepsis, which together account for up to 25% of SBO-related mortality [2]. Surgical intervention is indicated in cases of clinical deterioration despite conservative management, evidence of peritonitis, and signs of bowel ischemia or strangulation. Strangulation may present with fever, tachycardia, severe abdominal pain, and leukocytosis. The recurrence rate of SBO after initial surgical management ranges from 12% to 32% [3]. In contrast, functional SBO (ileus) is typically managed non-operatively, with emphasis on bowel rest, correction of metabolic abnormalities, minimizing opioid use, and use of prokinetic agents such as metoclopramide, when appropriate. 

Large bowel obstruction (LBO), though less common, poses a similarly urgent threat, particularly when associated with malignancy, volvulus, or strictures. Colorectal cancer remains the leading cause of mechanical LBO [6]. In both SBO and LBO, timely diagnosis and management are critical to prevent complications such as strangulation, ischemia, or perforation. Imaging, especially computed tomography (CT scan), plays a central role in diagnosis, while management strategies depend on underlying etiology, patient stability, and severity of obstruction [7]. Laboratory evaluation for LBO should include a CBC, anemia panel, renal function tests, ABG analysis, and screening for infections such as *Clostridium difficile*. Abnormal findings such as leukocytosis or metabolic acidosis may signal bowel ischemia and require urgent attention. Imaging is critical in diagnosing LBO. The initial imaging modality is usually an abdominal radiograph, in supine and upright or lateral decubitus position, which may show colonic dilation, a transition point, air-fluid levels, pneumoperitoneum, or an absence of gas distal to the obstruction. 

Colonoscopy can also be a valuable diagnostic tool, especially when there is a need for tissue biopsy. In situations where colonoscopy cannot be performed or is incomplete, the European Society of Gastrointestinal Endoscopy and the European Society of Gastrointestinal and Abdominal Radiology recommend alternative diagnostic approaches such as CT colonography or colon capsule endoscopy [8]. Management of patients with LBO depends on the etiology. However, there are some key principles of management that overlap between etiologies. Most patients with LBO have volume depletion and electrolyte imbalances. Therefore, intravenous fluid resuscitation and repletion of electrolytes are among the initial steps in management. In patients with signs of sepsis, ischemia, or perforation, antibiotics should be initiated [6,7]. LBO of any etiology presenting with these symptoms is associated with significantly higher mortality. For this reason, prompt surgical intervention is recommended. 

Intestinal obstruction is a common surgical emergency but becomes particularly challenging when caused by overlapping pathologies such as an obstructing sigmoid mass and dense intra-abdominal adhesions in a patient without prior abdominal surgery. Distinguishing between benign adhesive disease and malignancy can be difficult, especially in the setting of incomplete tissue diagnosis and ongoing infection or obstruction. This case highlights the diagnostic and therapeutic complexities encountered in a young patient with a large sigmoid mass causing combined SBO and LBO complicated by extensive adhesions and intra-abdominal inflammation. It also highlights how a sigmoid mass and delays in interventions can complicate the management of intestinal obstruction. Through a brief review of management strategies available, including surgical options and their associated risks and benefits, we aim to highlight approaches that may lead to improved outcomes in similar cases.

## Case presentation

We present the case of a 35-year-old male with no prior medical history and no family history of cancer who presented with poor oral intake along with nausea and vomiting. The patient denied any past surgeries and was not taking any medications. The patient did not complain of irregular bowel habits. He initially presented to an outside hospital with obstructive symptoms, including nausea, bilious vomiting, and obstipation. A CT scan demonstrated a large sigmoid mass causing bowel obstruction with concern for intestinal perforation. 

Outside hospital course

Day 0

A CT of the abdomen and pelvis demonstrated a large sigmoid colon mass causing bowel obstruction, with findings concerning for intestinal perforation. The patient underwent diagnostic laparoscopy with evacuation of ascites. No bowel resection or endoscopic evaluation was performed at that time.

Approximately Day 30

Because of persistent obstruction, the patient subsequently underwent exploratory laparotomy with creation of a transverse loop colostomy. The operating surgeon identified the sigmoid mass but elected not to resect it because of ongoing intra-abdominal infection and abscess formation. Percutaneous drains were placed by interventional radiology for management of the intra-abdominal abscesses. According to the outside hospital operative report, there was no gross evidence of metastatic disease.

The patient continued to have obstructive symptoms and infectious complications. He tolerated minimal oral intake and had very little bilious output from the colostomy. A nasogastric tube was placed for persistent nausea and vomiting, and total parenteral nutrition was initiated.

Approximately Day 60

The patient was transferred to our institution for a higher level of care. At the time of transfer, he had not undergone colonoscopy and did not yet have a tissue diagnosis.

Presentation to our institution

Laboratory evaluation demonstrated leukocytosis (21.4 ×10³/µL), normocytic anemia (hemoglobin 7.5 g/dL), hypokalemic hypochloremic metabolic alkalosis consistent with prolonged nasogastric decompression, a mildly elevated serum lactate (2.1 mmol/L), and an elevated carcinoembryonic antigen (CEA) level of 13.0 ng/mL (Table 1). A multidisciplinary decision was made to take the patient to the operating room for an exploratory laparotomy. 

**Table 1:** Pertinent laboratory investigations on admission following transfer to our institution. The values listed in bold are the abnormal lab values.

Parameter	Patient Value	Reference Range
White blood cell count (×10³/µL)	21.4	4.0–11.0
Hemoglobin (g/dL)	7.5	13.5–17.5
Hematocrit (%)	23.2	41–53
Platelet count (×10³/µL)	382	150–450
Sodium (mmol/L)	138	135–145
Potassium (mmol/L)	2.9	3.5–5.0
Chloride (mmol/L)	91	98–107
Bicarbonate (mmol/L)	36	22–29
Blood urea nitrogen (mg/dL)	20	7–20
Creatinine (mg/dL)	0.8	0.7–1.3
Glucose (mg/dL)	102	70–110
Calcium (mg/dL)	8.8	8.5–10.5
Magnesium (mg/dL)	2.0	1.7–2.4
Phosphorus (mg/dL)	3.5	2.5–4.5
Lactate (mmol/L)	2.1	0.5–2.0
Albumin (g/dL)	2.8	3.5–5.0
Carcinoembryonic antigen (CEA) (ng/mL)	13.0	<3.0 (nonsmoker)

Intraoperative Findings

Upon entering the abdomen, it was found that the entirety of the small bowel adhered to itself along with the abdominal wall. Several loops of bowel adhesions causing multiple separate closed-loop obstructions were found, and we were unable to decompress the small bowel. The colon, liver, and spleen could not be visualized due to the extensive adhesions of the large and small bowel fused to the peritoneum. During adhesiolysis, multiple nodules were noted in the mesentery as well as nodules that appeared to be in the small bowel lumen (Figure 1). 

**Figure 1:**
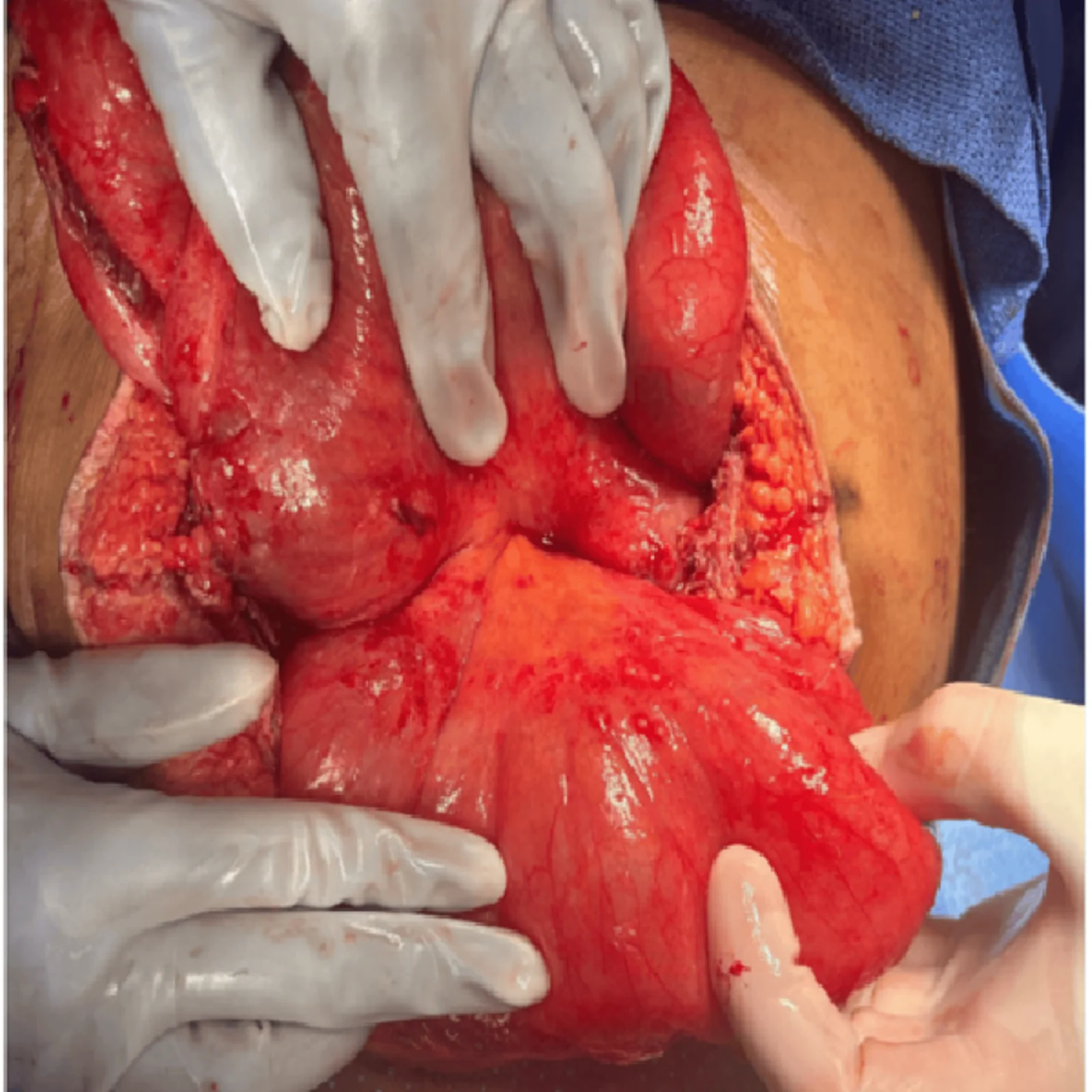
Intraoperative photograph demonstrating multiple mesenteric nodules encountered during exploratory laparotomy.

Biopsies of the mesenteric nodules and inflammatory tissue were obtained. Frozen section analysis of the mesenteric nodules was negative for malignancy. After several hours of adhesiolysis with minimal progress, a multidisciplinary discussion was held intraoperatively between trauma attendings, acute care surgery faculty, and colorectal surgery attendings. It was determined that given the extensive disease burden and hostile abdomen, further exploration and resection were considered futile. A palliative percutaneous endoscopic gastrostomy tube was placed for venting and comfort, allowing for oral intake and removal of the nasogastric tube.

The fascia was closed, and the skin was approximated with staples. An incisional wound vac was placed. A flexible sigmoidoscopy was performed and demonstrated a completely obstructing sigmoid mass approximately 35 cm from the anal verge that was unable to be traversed (Figure 2). Biopsies were obtained with cold forceps and sent for pathology evaluation. The patient was taken back to the post-anesthesia care unit in stable condition.

**Figure 2:**
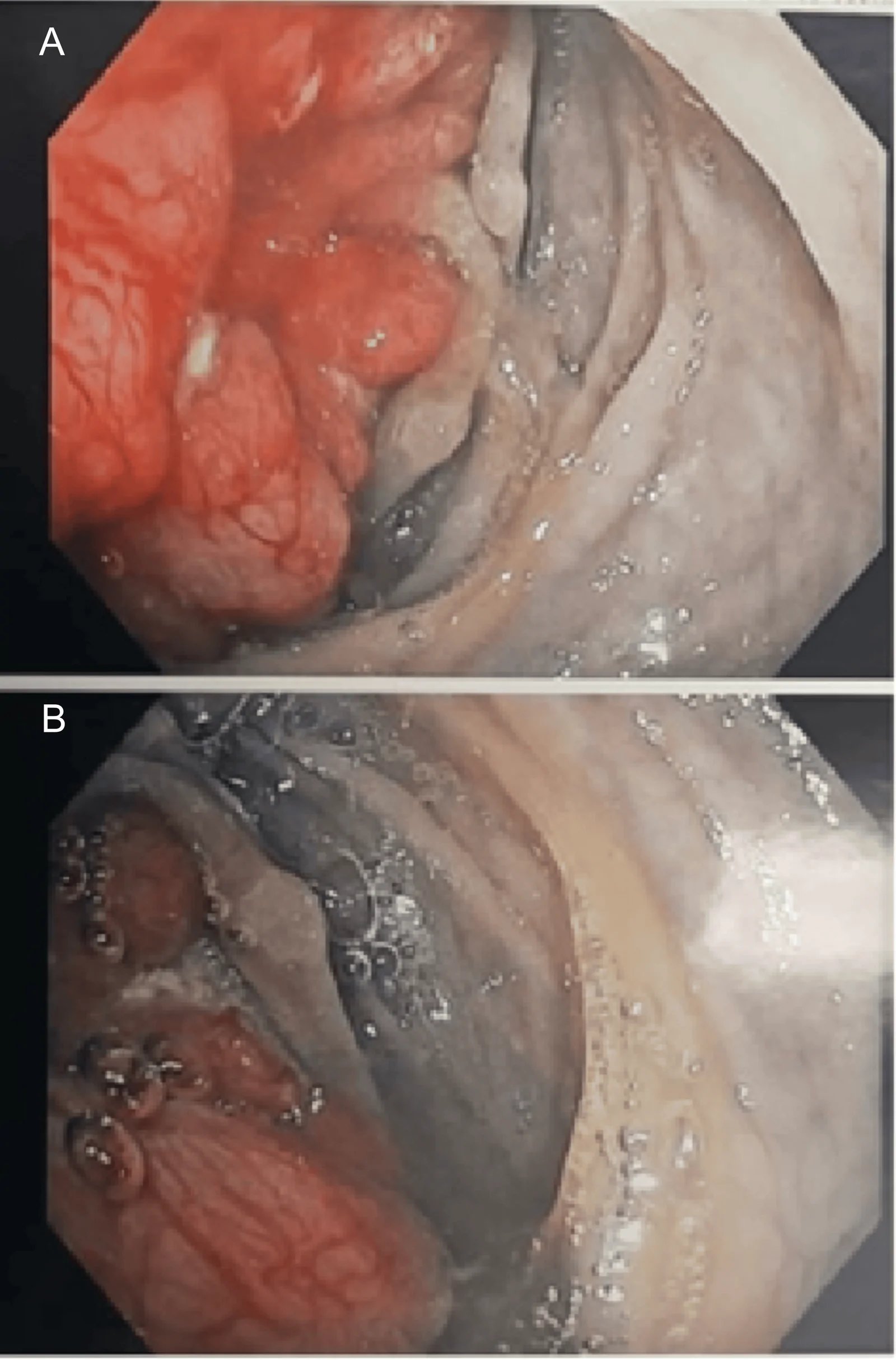
Flexible sigmoidoscopy demonstrating a completely obstructing sigmoid mass approximately 35 cm from the anal verge. A: close endoscopic view demonstrating the irregular, erythematous, lobulated surface of the mass; B: alternate view demonstrating severe luminal narrowing and the absence of a traversable lumen.

Postoperative Course and Pathological Diagnosis

Final pathology of the peritoneal nodules demonstrated fibrotic fibroconnective tissue with acute-on-chronic inflammation and a giant-cell reaction, without evidence of malignancy. Biopsy of a mesenteric lesion similarly demonstrated fibroadipose tissue with chronic inflammation and a giant-cell reaction, without malignancy.

The initial biopsy of the obstructing sigmoid mass obtained during flexible sigmoidoscopy demonstrated fibroadipose tissue with acute-on-chronic inflammation and a giant-cell reaction but no malignancy. Because these findings were discordant with the endoscopic appearance and clinical suspicion for an obstructing colorectal malignancy, repeat flexible sigmoidoscopy with additional biopsies was performed postoperatively. The repeat biopsies revealed moderately differentiated invasive adenocarcinoma. Cytologic evaluation of the ascitic fluid demonstrated rare atypical cells, multinucleated giant cells, acute inflammatory cells, and red blood cells, further supporting the diagnosis. Goals of care discussions were held with family. The family subsequently elected for palliative and comfort measures. 

## Discussion

This case highlights how discordance between a strongly malignant clinical picture and initially benign histopathology, followed by delayed definitive evaluation, can allow an obstructing colorectal cancer complicated by infection to progress into a surgically hostile abdomen in which definitive oncologic resection is no longer feasible. Three issues were central: the discrepancy between the elevated CEA level of 13.0 ng/mL and the obstructing mass versus the benign giant cell reaction reported on the initial biopsy; the dense inflammatory adhesions that developed in the setting of persistent obstruction and intra-abdominal abscess; and the absence of a prompt tissue diagnosis and definitive oncologic plan after fecal diversion.

Diagnosis of obstructive sigmoid mass according to current guidelines

Diagnosing LBO usually begins with supine and upright or decubitus X-rays, showing a dilated colon with a transition point in the area of the obstruction and a lack of gas distal to the area of obstruction. Air-fluid levels and pneumoperitoneum can also be observed on the X-rays, indicating perforation. However, Annals of Laparoscopic and Endoscopic Surgery reported that a CT scan had the highest sensitivity and specificity for diagnosing LBO in patients who are stable with signs of impending perforation [9]. Colonoscopy should be performed and is the gold standard for diagnosing LBO secondary to malignancy. It can provide details on the location and severity of obstruction, tissue biopsies can be taken, and the entire colon can possibly be assessed. If colonoscopy is not feasible due to complete obstruction or poor preparation, CT colonography or positron emission tomography (PET) could be beneficial and guide operative planning. 

Despite the advances in diagnosing suspected malignant LBO, many imaging techniques can only be performed in patients who are stable without any signs of perforation or infection. Our patient initially presented with obstructive signs with subsequent infection and re-obstruction, restricting his ability to undergo colonoscopy or colonography. 

Management of an obstructive sigmoid mass according to current guidelines

The first step in the management of a malignant LBO is determining the hemodynamic stability of the patient. If it is not acute, the patient should undergo complete staging before determining operative management. If the patient is hemodynamically unstable and requires immediate surgery, then they are categorized into one of three groups: high, moderate, and low surgical risk (Table 2) [7,10,11].

**Table 2:** Management of LBO in hemodynamically unstable patients by surgical risk. LBO: large bowel obstruction

Surgical Risk Level	Recommended Procedure
Low	Resect with primary anastomosis
Moderate	Resect with primary anastomosis or anastomosis and proximal diversion
High surgical risk with resectable tumor	Resect with end ileostomy or colostomy
High surgical risk with a difficult resection (T4 or rectal location)	Proximal diverting loop ostomy

Furthermore, the three goals of surgical management of LBO are decompression of the bowel, primary resection adhering to oncologic principles, and restoration of intestinal continuity. Given that, the first step in LBO management would be a nasogastric tube for decompression and fluid and electrolyte resuscitation. In addition, preoperative stoma marking and early nutritional assessment for postoperative parenteral/enteral support have proven to be beneficial. Our patient has a malignant LBO, and management of this etiology depends on the location of the tumor, patient factors, surgeon’s expertise, and available resources. 

There are two-stage and three-stage operative options for malignant bowel obstructions. In the two-stage option, the first surgery involves resection of the cancer-containing segment and primary anastomosis with diverting loop ileostomy or resection with end colostomy (Hartmann procedure). The second stage is reestablishing bowel continuity by taking down the ostomy. 

In the three-stage option, the first surgery is creating a diverting loop stoma or self-expanding metallic stents (SEMS), allowing for neoadjuvant therapy. SEMS is a minimally invasive procedure that serves as a bridge to surgery or palliative treatment in unresectable malignant colorectal obstruction [12]. It allows for prompt decompression of the colon and avoids the risks of emergency surgeries and stomas that are not reversed. Although SEMS has a success rate of 70-80%, its use should be cautioned in long-term management due to the risk of stent erosion, migration, and reconstruction over time [12]. SEMS should also not be placed in areas of acute angulation such as flexures due to the higher risk of stent erosion, migration, pain, and tenesmus. When SEMS is used as a bridge to surgery, it is typically followed by definitive resection and primary anastomosis. This brings us to the second stage, which is resection of the cancer-containing segment with proximal diversion. The third stage reestablishes intestinal continuity. 

Our patient presented with small bowel perforation and signs of LBO secondary to sigmoid mass, which limited the use of many less invasive diagnostic methods such as colonoscopy or CT colonography. Therefore, he underwent a diagnostic laparoscopy and transverse loop colostomy after CT imaging. Although the initial biopsy showed a benign giant cell reaction, that result was discordant with both the obstructing mass and the CEA level of 13.0 ng/mL and should not have been considered reassuring. Marked inflammation, ulceration, necrosis, and a foreign-body-type giant cell response may dominate a small superficial specimen while an underlying malignancy remains unsampled. In this setting, clinicopathologic discordance should prompt direct communication with pathology and repeat tissue acquisition through multiple biopsies, including deeper samples from viable areas of the lesion when safely feasible. During the initial laparoscopy, an intraoperative biopsy or frozen section analysis could have also helped to establish malignancy and guide staging and oncological management. Moreover, dense adhesions in a patient without prior abdominal surgery should have heightened concern for an aggressive malignant or inflammatory process. Cytologic analysis of the ascitic fluid collected intraoperatively could have provided insights into metastatic staging [13]. 

The subsequent "frozen abdomen" can be understood as the cumulative result of persistent mechanical obstruction and uncontrolled intra-abdominal infection. Ongoing bowel distention, local ischemic injury, perforation or leakage, and abscess formation produce intense peritoneal inflammation with fibrin-rich exudate. When this process persists, fibrin organization and fibroblast ingrowth convert initially separable inflammatory attachments into dense, vascular adhesions that can obliterate normal tissue planes. In such a hostile field, attempts at en bloc oncologic resection may carry prohibitive risks of enterotomy, hemorrhage, ureteral or vascular injury, and incomplete cancer clearance; in this patient, the inflammatory burden ultimately precluded a safe definitive resection [13].

A diverting colostomy was an appropriate acute maneuver to decompress the obstructed colon and reduce the immediate risk of further contamination, but it should have been treated as the first stage of a time-limited oncologic strategy rather than definitive management. Once sepsis was controlled and the patient stabilized, the discordant pathology required urgent repeat or deeper biopsy, completion of staging, and multidisciplinary planning for definitive resection. In this case, a plan for diagnostic confirmation and reassessment for oncologic resection within approximately two to three weeks would have limited open-ended delay while allowing initial physiologic optimization. Failure to secure a tissue diagnosis and establish that plan permitted persistent obstruction, abscess formation, and progressive inflammatory adhesions, eventually converting a potentially manageable lesion into an unresectable hostile abdomen. This interval is a case-specific target, not a universal guideline; timing must reflect sepsis control, nutritional status, operative risk, and local oncologic resources.

## Conclusions

In conclusion, our case report aims to highlight the importance of timely diagnosis, definitive surgical management, and coordinated multidisciplinary planning, even in emergent or infected settings. The lack of definitive resection or tissue diagnosis, coupled with persistent proximal obstruction and infection, ultimately led to a poor patient prognosis. 
